# Assessment of Different Strategies for Composting of the Two-Phase Olive Mill Solid Waste: A Demonstrative Scale

**DOI:** 10.1021/acsagscitech.5c00286

**Published:** 2025-08-11

**Authors:** Sara Velilla-Delgado, Juan Cubero-Cardoso, Antonio Serrano, Elisabet Aranda, Concepción Calvo, Tatiana Robledo-Mahón

**Affiliations:** † Environmental Microbiology Group, Institute of Water Research, 16741 University of Granada, Espacio V Centenario, Avenida Madrid 11, 18012 Granada, Spain; ‡ Department of Microbiology, Pharmacy Faculty, University of Granada, Campus Universitario Cartuja s/n, 18011 Granada, Spain

**Keywords:** aeration, alperujo, bioaugmentation, biodegradation, manure, phenolic compounds, phytotoxicity, semipermeable membrane

## Abstract

Thousands of tons of two-phase olive mill solid waste (2P-OMSW) are generated annually, necessitating effective valorization strategies. Composting has been widely explored as a management approach; however, the extended processing time required for these residues poses a significant challenge for the olive industry. In this study, a forced aeration system combined with a semipermeable cover was implemented at a demonstrative scale to enhance the composting process and reduce its duration. Additionally, process optimization was evaluated through a two-stage composting strategy. In stage I, compost preconditioning was carried out using two types of manure (poultry and cow). In stage II, a bioaugmentation process was introduced using the edible fungus *Pleurotus eryngii*. The composting of 2P-OMSW under forced aeration and a semipermeable cover lasted 90 days. During the composting process, physicochemical parameters, total phenol content, microbial analysis, and phytotoxicity bioassays were measured to evaluate the efficiency and quality of the final compost. In stage I, poultry manure proved to be more effective than cow manure, resulting in a lower C/N ratio (<25%), higher nitrogen, phosphorus, and potassium content, and a greater reduction in total phenol content (>70%). In stage II, bioaugmentation significantly enhanced the removal of heavy metals, particularly zinc (Zn) and copper (Cu). Both final composts, obtained within 90 days, exhibited enriched nutrient content, stabilized nonphytotoxic organic matter, and low heavy metal concentrations. The findings highlight the potential of a forced aeration system combined with a semipermeable cover as an effective strategy for composting 2P-OMSW. This approach facilitates the transformation of 2P-OMSW into high-quality compost, making it suitable for use as an organic amendment or fertilizer in agricultural systems. Furthermore, it allows for the management of this residue within a relatively short time frame.

## Graphical abstract

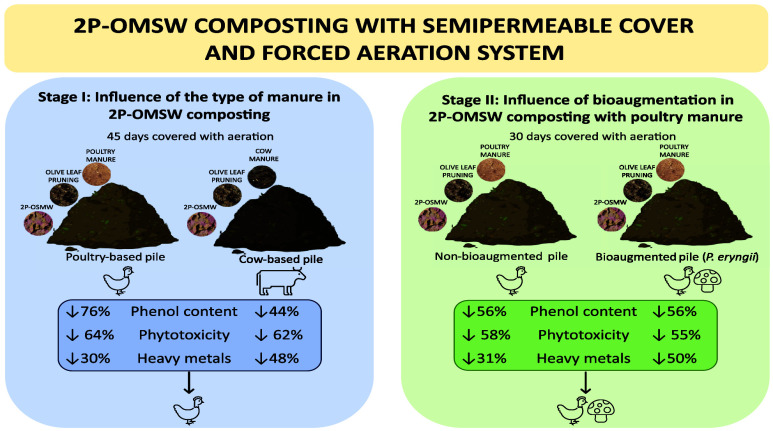

## 1 Introduction

The agro-industrial sector produces large amounts of biodegradable waste and byproducts that must be properly disposed of to reduce environmental impact.^1^ A clear example is the olive oil industry, a major sector in the Mediterranean region, which generates significant quantities of organic waste, both liquid and solid, depending on the extraction method used.^2^ These substrates are considered a potential source of environmental pollution and present a serious management challenge for olive oil-producing countries.^3^ In Spain, the main byproduct of olive oil mills is two-phase olive mill solid waste or 2P-OMSW, produced through the widespread use of the two-phase centrifugation system.^4^ Annually, approximately 4 million tons of 2P-OMSW are produced in the country.^2,4^ The centralization of processing this solid waste by the pomace extract industries, primarily for pomace oil extraction, is a key advantage in Spain.^5^ However, the increasing production volume, the high drying costs, and the growing interest in the bioactive compounds present in this byproduct are driving the development of improved alternatives or combined management strategies.^5^

2P-OMSW is a lignocellulosic material with a high moisture content (approximately 60–70%), making it difficult to handle and transport.^6^ It contains potentially polluting and phytotoxic compounds, such as phenols and fats.^7^ Various methods for 2P-OMSW treatment have been explored, such as extraction of bioactive compounds, biogas production, bioethanol production, volatile fatty acid production, composting, etc.^3,8^ Organic byproducts should be reused as a source of organic matter and nutrients, minimizing carbon footprint and input costs by recycling, valorization, and returning them to the agricultural system from which they were obtained.^8^ They are particularly valuable in organic farming, where organic matter and macronutrient sources are limiting factors for production. Recycling agricultural and livestock waste through composting as organic amendments in soils with intensive and low-organic-matter crops is an important environmental strategy.^9^ Composting promotes the biotransformation of raw organic matter into a more stable and complex product enriched with humic substances.^9^ The application of compost helps restore degraded soils by increasing organic matter content and enhancing physical, chemical, and biological properties.^10^ This practice can also mitigate soil degradation issues related to declining organic matter in agricultural soils in the Mediterranean region. Applying compost improves soil quality and fertility by increasing organic matter content and the long-term bioavailable fractions of nitrogen, phosphorus, and potassium.^2^ Further, several studies have shown that cocomposting olive oil mill byproducts with complementary substrates (such as pruning residues and animal manure) could be an effective recycling option.^2,3,6,11,12^

Regarding composting systems, there are different options based on technological complexity, which often lead to more efficient processes but also to higher investment and/or operational costs. Traditional methods involve the mechanical turning of compost piles to facilitate aeration throughout the process. Using this approach, composting times for 2P-OMSW typically range from six to eight months.^12–15^ Among innovative composting techniques with no high investment and operating costs, notable options include static piles with active system floor aeration and semipermeable covers. In these systems, the composting matrix is crossed by pipes that insufflate air, ensuring an adequate supply of oxygen to support the aerobic biological processes essential to composting.^16^ Although forced aeration systems improve composting over passive aeration, studies have shown that adding semipermeable membranes to composting systems offers significant benefits in both composting efficiency and operational cost compared to forced aeration systems without cover.^17–19^ These semipermeable membranes are layers of laminated fabric consisting of three membranes with micropores between two robust, breathable textiles.^20^ This design provides high water resistance, helps maintain humidity within the pile, and prevents heat loss, which therefore helps maintain process temperatures more effectively. Additionally, these covers may contribute to reducing odor emissions, reducing the environmental impact during composting.^6,20^ Furthermore, composting systems can be bioaugmented with microorganisms capable of degrading specific components, such as phenolic compounds found in 2P-OMSW. In this context, fungi are the most interesting microorganisms for this purpose due to their abilities to produce extracellular enzymes to degrade recalcitrant substances like lignin or phenols, biodegrade microcontaminants like pharmaceuticals and microplastics, and adsorb heavy metals effectively.^21^ Additionally, fungi can synthesize substances that promote plant growth, which may increase the agricultural value of the final compost product. When selecting bioaugmentation for composting enhancement, it is essential to consider operational improvements as well as human health and environmental safety, avoiding pathogenic fungi or species that could pose a risk to the application area.^22^

Based on the previous considerations, this study aimed to evaluate the composting performance of 2P-OMSW using static demonstrative pile models with forced aeration under a semipermeable cover. In this system, olive leaf pruning was used as a bulking agent, 2P-OMSW as the primary composting material, poultry and cow manures as an amendment, and a bioaugmented (*Pleurotus eryngii*). The research methodology was designed in two stages. In stage I, the effectiveness of adding two types of manures, poultry and cow, was compared. In the second stage, the effectiveness of the bioaugmentation process with *P. eryngii* was evaluated.

## 2 Materials and Methods

### 2.1 Demonstrative Experimental Setup and Composting Materials

A linear composting system, with a capacity for piles of a size of 10 m (length) × 3 m (width) × 2 m (height), was built at the facilities of the olive oil mill Oleomontes SCA located in Torrecardela, Granada, Spain (geographic coordinates: 37.49135298257381, −3.3457714610046194). To test the different conditions, we utilized the same cover and piping system. The system was divided into two piles of approximately 4 m each, separated by an intermediate 1 m zone. This design allowed for the evaluation of two conditions in each stage. Each pile was composed of 2P-OMSW, as waste to be composted, and olive leaf pruning as a bulking agent. Both were supplied by Oleomontes SCA, and poultry and cow manures as amendments were supplied by Reniego SL, a local company (geographic coordinates: 37.361380, −3.282868). The physicochemical characterization of starting materials (2P-OMSW, both manures and olive leaf pruning) is shown in Table 1.

**Table 1:** Physicochemical Characterization of Starting Materials of Stages I and II^a^,^b^

	stage I				stage II		
	2P-OMSW	olive leaf pruning	poultry manure	cow manure	2P-OMSW	olive leaf pruning	poultry manure
pH	4.3 ± 0.1	6.3 ± 0.7	9.1 ± 0.1	8.6 ± 0.1	6.7 ± 0.2	6.7 ± 0.1	7.3 ± 0.2
EC (dS/m)	0.9 ± 0.1	0.6 ± 0.1	2.5 ± 0.1	1.4 ± 0.1	0.9 ± 0.1	1.1 ± 0.1	3.1 ± 0.1
TS (% w/w)	18.0 ± 0.1	79.0 ± 1.0	86.5 ± 3.5	62.0 ± 1.4	42.0 ± 0.1	50.0 ± 0.1	67.0 ± 1.0
VS (% w/w)	16.0 ± 0.1	72.3 ± 0.6	39.5 ± 0.7	47.0 ± 1.4	37.3 ± 0.1	43.0 ± 0.1	56.3 ± 0.6
humidity (%)	82.1 ± 0.2	21.1 ± 0.6	13.3 ± 3.5	37.8 ± 1.6	58.0 ± 0.4	49.9 ± 0.2	32.7 ± 0.8
C (% w/w)	9.2 ± 0.1	42.0 ± 0.4	22.9 ± 0.2	27.4 ± 0.7	21.4 ± 0.4	25.1 ± 0.2	32.4 ± 0.2
N (% w/w)	0.3 ± 0.1	0.9 ± 0.0.1	1.8 ± 0.1	1.4 ± 0.1	0.6 ± 0.1	0.8 ± 0.1	2.9 ± 0.1
C/N	35.1 ± 1.0	47.0 ± 1.9	12.9 ± 0.8	20.3 ± 1.5	33.7 ± 1.2	30.4 ± 0.8	11.2 ± 0.3
P_2_O_5_ (% w/w)	0.1 ± 0.1	0.2 ± 0.1	1.3 ± 0.1	1.4 ± 0.1	0.1 ± 0.1	0.2 ± 0.1	1.2 ± 0.1
K_2_O (% w/w)	0.7 ± 0.1	0.4 ± 0.1	2.5 ± 0.2	0.8 ± 0.1	0.7 ± 0.1	0.9 ± 0.1	2.2 ± 0.1
CaO (% w/w)	0.1 ± 0.1	1.9 ± 0.1	9.9 ± 1.1	4.5 ± 0.2	1.1 ± 0.1	2.1 ± 0.1	1.5 ± 0.1
MgO (% w/w)	0.1 ± 0.1	0.3 ± 0.1	1.7 ± 0.1	1.0 ± 0.1	0.2 ± 0.1	0.3 ± 0.1	0.5 ± 0.1
Na_2_O (% w/w)	0.02 ± 0.0.1	0.04 ± 0.01	0.43 ± 0.02	0.39 ± 0.1	0.1 ± 0.1	0.1 ± 0.1	0.3 ± 0.1
total phenols (g gallic acid eq./kg)	5.5 ± 0.1	17.4 ± 1.4	1.2 ± 0.1	2.4 ± 0.1	7.5 ± 0.2	8.0 ± 0.6	9.6 ± 0.0.3
GI (%)	2.58 ± 0.38	96.61 ± 5.01	57.32 ± 5.58	95.08 ± 4.70	1.03 ± 0.17	3.27 ± 0.33	0.80 ± 0.18

a All values were obtained in triplicate; the mean and standard deviation values are included.

b Conductivity (EC), total solids (TS%), mineral solids (MS%), volatile solids (VS%), total organic matter of dry sample (TOM%), total organic carbon (C%), total nitrogen (N%), germination index (GI%).

Oxygen supply (aeration) was provided through a forced aeration system, where air was injected into the base of the pile. Aeration was set to operate actively for 2 min every 10 min, at a rate of 20 m^3^/h. To ensure uniform air distribution across the entire base of the pile, prevent undesired anaerobic conditions, and avoid clogging of the air outlet holes, the area of the air injection conduits was covered with almond shells, forming a 20–30 cm thick layer. The composting experiment lasted 90 days, from April to July 2024 in stage I and from June to September in stage II (Table 2). In stage I, we compared the application of poultry and cow manure, and in the second stage, the effect of bioaugmentation by *P. eryngii* using the selected manure from stage I. The bioaugmentation was carried out using fragments of approximately 15 g of mycelia of the fungus *P. eryngii*, distributed at 8 equidistant points of the pile at a depth of 30 cm, inoculated on days 7 and 30 of the composting process. This fungus was purchased from the company “Cultivos Forestales y Micológicos S.L.” under the name “Micecardo: mycelium of King trumpet mushroom.”

**Table 2:** Composting Process Parameters and Treatment Conditions

	materials (% w/w)							
	2P-OMSW	olive leaf pruning	manure	covered time (day)	maturation time (day)	turning (day)	irrigation (day)	bioaugmentation (day)
Stage I								
poultry-based pile	60	30	10	45	45	30	30	
cow-based pile	50	30	20	45	45	30	30	
Stage II								
nonbioaugmented pile	60	30	10	30	60	30	30	
bioaugmented pile	60	30	10	30	60	30	30	7 and 30

### 2.2 Analytical Determination

#### 2.2.1 Sampling

Sampling was performed on days 0, 15, 30, 45, and 90, using the sampling protocol according to the composite sample procedure. For that, sampling was carried out in five equidistant sites at different depths within each pile (0, 25, 50, 75, and 100 cm) and mixed to create a single sample. Following the acquisition, each composite sample was transported at 4 °C, and subsamples for each sample were stored at 4 °C for the analysis performed on the same day and at −20 °C for further analyses.

#### 2.2.2 Physicochemical Determination

Pile temperature was monitored daily using a portable sensor at two core locations. Physiochemical parameters, including conductivity (EC), pH, humidity (%), total solids (TS%), mineral solids (MS%), volatile solids (VS%), total organic matter of dry sample (TOM%), total organic carbon (C%), total nitrogen (N%), C/N ratio, P_2_O_5_ (%), K_2_O (%), CaO (%), MgO (%), Na_2_O (%), and heavy metals (Cu, Cd, Cr, Ni, Pb and Zn in mg/kg), were assessed. Samples were characterized using standard methods for compost analysis from the Test Methods for the Examination of Composting and Compost, or “TMECC.”^23^ All analyses were carried out in triplicate.

#### 2.2.3 Total Phenol Content

The total phenol content was quantified after a solid–liquid extraction of the samples in accordance with Thompson et al.^24^ For that, 20 mL of methanol–water (v/v, 80/20) was added to 10 g of the solid or liquid fraction and incubated for 1 h at 70 °C in a water bath and then microfiltered with 0.2 μm nylon microfilters.^24^ The total phenol content was then determined in the obtained liquid fraction by the Folin–Ciocalteu (PanReac AppliChem, Spain) spectrophotometric method and expressed as grams of gallic acid equivalents per kilogram of sample.^25^

#### 2.2.4 Bacterial Pathogen Detection

For the detection of *Escherichia coli*, the standardized presence/absence procedure outlined in ISO 16649–1/2/3:2001 was followed using TBX medium (Tryptone Bile X-Glucuronide Agar, ref 4021562, Sigma-Aldrich). *Salmonella* spp. analysis was conducted in accordance with ISO 6579–1:2017, using RBSD medium (Rappaport Vassiliadis *Salmonella* Enrichment Broth, ref 4019792, Sigma-Aldrich) and XLD agar (Xylose Lysine Deoxycholate Agar, ref 4022062, Sigma-Aldrich).

#### 2.2.5 Isolation and Identification of Composting Fungi

Fungi present in the compost piles were isolated through serial dilutions on a malt extract agar (MEA, Biolife, Italy) medium with 50 mg/L streptomycin sulfate and tetracycline hydrochloride (Thermo Fisher Scientific), which was incubated at 28 °C for 5–7 days. According to the diversity and morphology of the fungal colonies that grew on the surface of the medium, selected morphotypes were transferred to a new MEA plate for isolation. Fungal isolates were sent to the Instrumental Techniques Laboratory of the University of León for their identification. Briefly, DNA was extracted, and the intergenic region and 28S rRNA gene were used for amplification and sequencing according to the methodology previously described by Robledo-Mahón et al.^26^ The obtained sequences were then compared to the GenBank (National Centre for Biotechnology Information (NCBI), http://www.ncbi.nlm.nih.gov).

#### 2.2.6 Phytotoxicity Bioassays

Phytotoxicity toward terrestrial plants was assessed using cress (*Nasturtium officinale*) seeds following the Zucconi protocol.^27^ Composite samples were diluted with distilled water (1:10 w/v) and shaken for 1 h at 120 rpm. Before the assays, the seeds were concurrently hydrated with tap water. Germination assays were conducted in 9 cm glass Petri dishes lined with filter paper containing 2 mL of each extract; a control with distilled water instead of an extract was also included. Twenty seeds were placed per plate and incubated for 48 h at 28 °C. The germination index, as described by Zucconi,^27^ was calculated using eqs 1–3 as follows

1 $$\%\mathrm{RSG}=\frac{G}{G_{0}}\times 100$$

2 $$\%\mathrm{RRG}=\frac{L}{L_{0}}\times 100$$

3 $$\%\mathrm{GI}=\frac{\%\mathrm{RSG}}{\%\mathrm{RRG}}\times 100$$

where RSG is relative seed germination, RRG is relative radicle growth, *G* is the number of germinated seeds with the sludge extract, *G*_0_ is the number of germinated seeds into the control dish, *L* is the length of the radicle in the seeds germinated with the sludge extract, and *L*_0_ is the length of the radicle in the seeds germinated into the control dish.

## 3 Results and Discussion

### 3.1 Stage I: Influence of the Type of Manure

This stage of the study analyzed the influence of poultry and cow manures on the main characteristics and the time of the composting process. After 4 days, the temperature ranged between 55 and 65 °C, indicating the start of the thermophilic stage, with a peak of 68 °C on day 11 regardless of the used manure (Figure 1). After 25 days, the temperature dropped to around 30 °C, showing the second mesophilic stage. Following irrigation and turning on day 30, the temperature in both piles remained below 30 °C. The temperature during stage I evolved similarly in both compost piles, regardless of the type of manure applied, with a thermophilic stage (temperature above 45 °C) observed from day 5 to day 25. The temperature was markedly higher than the ambient temperature recorded in the nearest meteorological station (Guadix, Granada), which ranged from 9 °C (minimum) to 32 °C (maximum). In stage I, the semipermeable cover was removed on day 45 to initiate the maturation stage. This duration of the composting process is much shorter than the one described by Varnero et al.,^28^ which reported a composting duration of 135 days for 2P-OMSW using forced aeration without cover. The difference could be attributed to the semipermeable cover and the use of poultry as a biostimulation strategy. In fact, Robledo-Mahón et al.^29^ described a reduction in the time required in the semipermeable cover period for the composting of sewage sludge at real scale from 60 to 30 days due to the implementation of semipermeable covers in combination with a forced aeration system on the floor. Moreover, Rueda et al.^30^ have reported that the use of animal manure may speed up the composting process since the nutrients provided by this can stimulate the microbial activity and, in consequence, degradation. Regarding pathogens, *Salmonella* spp. and *E. coli* were not detected after 90 days in any composting pile (Table 3). However, other results have reported that the use of poultry-based manure composts would not guarantee the removal of *E. coli*.^31^ In our study, the sustained temperatures above 55 °C for more than 5 days ensured proper sanitation during stage I.^32^

**Figure 1:**
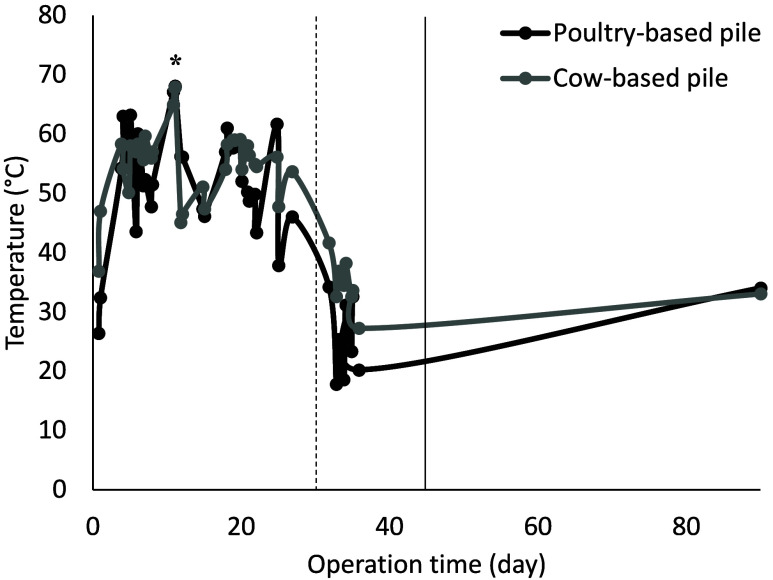
Temperature evolution during the composting process in the poultry-based pile and the cow-based pile. (−) Turning and irrigation, (*) maximum temperature reached, and (−) removal of semipermeable cover.

**Table 3:** Physicochemical Properties during Stage I of the Composting Process with Their Standard Deviation (±)^a^

poultry-based pile	*t*_initial_	15 days	30 days	45 days	90 days
pH	6.8 ± 0.2	7.8 ± 0.2	8.1 ± 0.1	7.7 ± 0.1	7.5 ± 0.1
EC (dS/m)	1.4 ± 0.2	1.5 ± 0.2	1.2 ± 0.0	1.7 ± 0.0	1.7 ± 0.1
humidity (% w/w)	42.7 ± 4.8	41.1 ± 1.7	39.2 ± 1.3	7.3 ± 0.2	7.2 ± 0.9
TS (% w/w)	57.3 ± 5.0	58.7 ± 1.5	60.7 ± 1.5	92.7 ± 0.6	93.0 ± 1.0
MS (% w/w)	10.9 ± 2.0	16.8 ± 2.5	14.4 ± 0.8	22.1 ± 1.8	24.3 ± 2.2±
VS (% w/w)	46.3 ± 3.8	42.0 ± 1.0	46.3 ± 2.1	70.3 ± 1.8	68.3 ± 2.5
TOM dry weight (% w/w)	80.9 ± 2.6	71.6 ± 3.6	76.3 ± 1.7	76.1 ± 2.0	73.8 ± 2.3
C (% w/w)	26.9 ± 2.2	24.5 ± 0.7	26.9 ± 1.1	40.9 ± 1.1	39.7 ± 1.3
N (% w/w)	0.9 ± 0.1	1.1 ± 0.1	1.1 ± 0.1	1.8 ± 0.0	1.7 ± 0.1
C/N	31.4 ± 1.6	22.2 ± 2.1	25.2 ± 1.2	23.0 ± 0.3	23.1 ± 1.4
*E. coli* (log CFU/g)					n.d
*Salmonella* spp. (log CFU/g)					n.d
cow-based pile	*t*_initial_	15 days	30 days	45 days	90 days
pH	6.9 ± 0.1	7.4 ± 0.1	8.1 ± 0.1	7.9 ± 0.1	7.3 ± 0.1
EC (dS/m)	1.3 ± 0.1	1.1 ± 0.0	0.9 ± 0.1	1.3 ± 0.1	1.4 ± 0.1
humidity (% w/w)	43.6 ± 3.6	40.6 ± 5.3	43.0 ± 2.5	13.2 ± 1.0	5.3 ± 0.4
TS (% w/w)	56.3 ± 3.1	59.0 ± 5.3	57.0 ± 2.6	86.7 ± 1.2	94.7 ± 0.6
MS (% w/w)	13.1 ± 0.4	10.2 ± 1.1	12.7 ± 1.9	20.9 ± 4.5	19.5 ± 1.
VS (% w/w)	43.3 ± 3.1	43.0 ± 5.2	44.3 ± 2.1	65.7 ± 5.5	75.3 ± 0.6
TOM dry weight (% w/w)	76.7 ± 0.6	82.0 ± 1.6	77.7 ± 2.3	76.0 ± 7.1	79.5 ± 0.9
C (% w/w)	25.1 ± 1.8	24.7 ± 2.8	25.7 ± 0.4	38.2 ± 3.2	43.6 ± 0.3
N (% w/w)	0.9 ± 0.0	1.0 ± 0.1	1.0 ± 0.1	1.5 ± 0.0	1.7 ± 0.0
C/N	28.5 ± 1.0	26.6 ± 5.5	26.3 ± 3.3	25.2 ± 1.9	26.0 ± 0.8
*E. coli* (log CFU/g)					n.d
*Salmonella* spp. (log CFU/g)					n.d

a n.d. Not detected, conductivity (EC), total solids (TS%), mineral solids (MS%), volatile solids (VS%), total organic matter of dry sample (TOM%), total organic carbon (C%), total nitrogen (N%).

The physicochemical parameters in the poultry-based pile and cow-based pile throughout the composting process are presented in Table 3. In the poultry-based pile, pH values increased slightly from 6.8 to 7.5 during the composting process; similarly, in the cow-based pile, they ranged from 6.9 to 7.3. Conductivity varied from 1300 to 1700 μS/cm in the poultry-based pile, whereas it remained stable at 1400 μS/cm in the cow-based pile. Both pH and conductivity values were within the described ranges for an optimal composting process.^33^ Initial humidity levels remained constant at 40% for the first 30 days, indicating that the material proportions were suitable for maintaining optimal humidity. However, after turning at day 30, humidity levels drastically dropped to 7% (poultry-based pile) and 13% (cow-based pile), as was detected in the 45-day sample. By day 90, final humidity levels were 7% in the poultry-based pile and 5% in the cow-based pile (Table 3). Total organic matter (TOM) content in dry weight decreased from 81 to 74% in the poultry-based pile, while no significant changes were observed for the cow-based pile (Table 3). TOM is a critical quality parameter, as composts are frequently used to enhance soil organic matter. For such applications, composts with a higher organic matter content are generally preferred.^33^

The nitrogen content gradually increased from 0.9 to 1.7% in both piles (Table 3). Similarly, the carbon content showed an upward trend, rising from 27 to 40% in the poultry-based pile and from 25 to 44% in the cow-based pile. Consequently, the C/N ratio decreased by 32% in the poultry-based pile, reaching a final value of 23, and by 9% in the cow-based pile, with a final value of 26. The final values of C/N ratios were higher than others previously described for compost mixtures including animal manures, probably due to the low nitrogen content of the 2P-OMSW, the main substrate to be composted in the present research (0.3 ± 0.1%).

The percentage of macronutrients throughout the operation time (Figure S1) showed a small increase in their percentage. The variations were similar to the losses of organic matter. Calcium was the most abundant determined macronutrient, reaching almost 7% in the poultry-based pile and 6% in the cow-based pile. In addition, potassium showed a percentage close to 2% at the end of the composting process in both piles (Figure S1). On the other hand, phosphorus, magnesium, and sodium at the end of the composting process had a percentage below 1% in both piles. These values fall within the typical ranges observed in compost, where phosphorus concentrations generally range from 0.2 to 1.3%, and potassium levels typically vary between 0.3 and 2.0%.^33^ The NPK ratio was 1.7/0.3/1.7 for the poultry-based pile and 1.7/0.3/1.4 for the cow-based pile. The results of the concentrations of heavy metals in each of the composting piles at the start and end of the process are shown in Table 4. The highest concentrations corresponded to Zn and Cu for both piles. The high initial presence of these metals may probably come from the olive leaf pruning. At the end of this stage, the total heavy metal concentration decreased by 30% in the poultry-based pile and 48% in the cow-based pile. The observed reduction in the metal content for both piles would be a consequence of the mobilization of the metals during the composting.^34^

**Table 4:** Concentration of Heavy Metals Expressed in mg/kg of the Poultry-Based Pile and the Cow-Based Pile in the Initial and Final Mixtures of the Composting Process with Their Standard Deviation (±)^a^

	poultry-based pile		cow-based pile	
	initial	final	initial	final
Hg (mg/kg)	<0.1	<0.1	<0.1	<0.1
Pb (mg/kg)	8.2 ± 6.6	4.2 ± 0.7	4.6 ± 1.8	2.3 ± 0.6
Ni (mg/kg)	7.5 ± 2.7	5.1 ± 0.7	9.9 ± 1.8	4.8 ± 1.5
Cd (mg/kg)	<0.5	<0.5	<0.5	<0.5
Cr (mg/kg)	9.4 ± 3.6	6.6 ± 1.6	14.7 ± 6.4	6.3 ± 3.3
Zn (mg/kg)	99 ± 21	71 ± 12	119 ± 5	59 ± 13
Cu (mg/kg)	77 ± 29	52 ± 11	79 ± 4	46 ± 1
Cr (VI) (mg/kg)	n.d	n.d	n.d	n.d

a n.d: Not detected.

During the composting process, the concentration of total phenol content was monitored due to its phytotoxic and antimicrobial potential effects.^12^ Figure 2 shows a clear decrease in the concentration of total phenols, particularly in poultry-based pile, where a reduction from 4.2 ± 0.3 g gallic acid equiv/kg of sample to 1.0 ± 0.1 g gallic acid equiv/kg of sample was observed, i.e., 76.2% reduction. In the case of cow-based pile, the percentage of total phenols content was reduced by 44.4%, from 3.6 ± 0.1 g gallic acid eq/kg of sample to 2.0 ± 0.1 g gallic acid eq/kg of sample on day 90. The phytotoxicity index, expressed as a germination percentage of cress seeds from each compost, is shown in Figure 2. The germination index of the seeds using compost from the two piles at the end of the composting process showed a value higher than 60%. An increase in the percentage of the germination index was observed after 30 days of composting, associated with a reduction in the concentration of total phenol content below 3 g of gallic acid eq/kg in both piles. Similar findings have been reported in other studies, indicating a correlation between lower GI values and the presence of phenolic compounds.^35,36^

**Figure 2:**
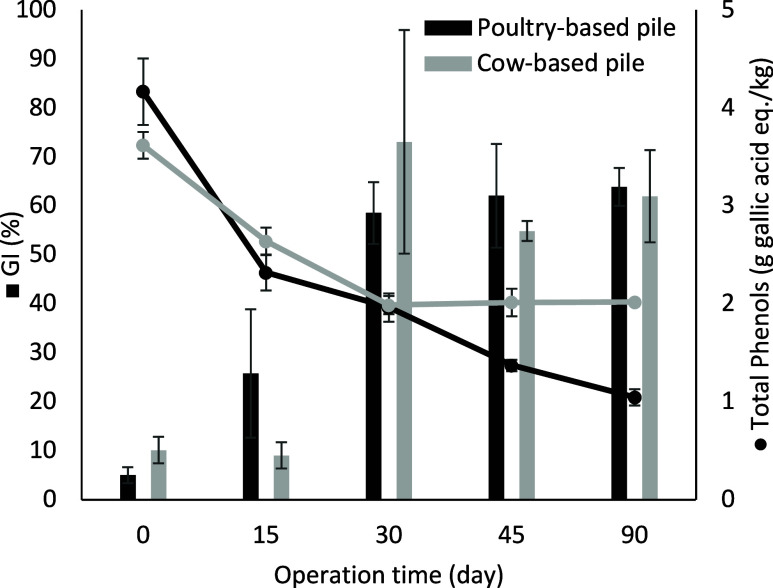
Germination index (GI%) (represented by bars) and total phenol content (depicted by points) for the poultry-based pile and the cow-based pile. Error bars represent the standard deviation of triplicate measurements.

The overall results showed that the use of poultry manure could be preferable to cow manure as an amendment, allowing a more favorable C/N ratio and total phenol content removal efficiency. Nevertheless, the composting of 2P-OMSW at a demonstrative scale was feasible in both poultry-based and cow-based piles, highlighting the flexibility of this technology and ensuring that adapting the initial conditions to the substrates available in each area does not necessarily limit its implementation.

During the removal of the cover, a high colonization of fungi on the surface of the composting pile was observed. Some samples of both piles from stage I were taken for fungal isolates (Table 5). Among the isolated fungi, *Aspergillus* and *Paecilomyces* were identified. In this sense, the observed reduction in total phenolic content may have been facilitated by *Paecilomyces variotii* and *Aspergillus terreus* (Table 5), which have been identified in various studies as microorganisms capable of degrading phenolic compounds and are regarded as potential biodetoxifiers.^37–39^

**Table 5:** Identification of Fungi and Yeasts in the Poultry-Based Pile and the Cow-Based Pile from Stage I

	fungi	%similarity	acc. number
poultry-based pile	*Aspergillus spinulosporus*	>99	NR_137459.1
	*Lichtheimia corymbifera*	>99	NR_111413.1
	*Ogataea polymorpha*	>99	KY102443.1
cow-based pile	*P. variotii*	>99	NR_130679.1
	*A. terreus*	>99	NR_131276.1
	*Lichtheimia ramosa*	>99	NR_111438.1
	*Yamadazyma* sp.	>99	KF666638.1

A strain belonging to *O. polymorpha* was identified in the poultry-based pile and another strain belonging to *Yamadazyma* genera in the cow-based pile. *O. polymorpha* is a yeast that has been previously detected in food waste composting.^40^ This species is well-known to tolerate high temperatures, grow fast, metabolize methanol, and be involved in nitrate fixation.^41^ This factor may have a positive effect on the compost value in the agricultural application of this compost. On the other hand, *Yamadazyme* sp. was isolated from the cow-based pile. This yeast has been previously detected in composting, and it is able to break down sugars to produce alcohol and carbon dioxide.^42^ Species belonging to this genus has been identified in olive mill waste.^43^

### 3.2 Stage II: Influence of Bioaugmentation with *P. eryngii* on the Quality of the Compost Produced

Taking into account the results of stage I, which included a lower C/N ratio, higher nutrient concentration, and a significant reduction in the total phenol content, stage II was strategically designed with poultry manure as a key process amendment, and olive leaf pruning served as the bulking agent. In this stage II, the effect of bioaugmentation with *P. eryngii* on the composting process and the quality of the final compost was evaluated. *P. eryngii* is an edible and commercialized fungi with a potent set of ligninolytic enzymes (laccase, Mn-oxiding peroxidases, and aryl-alcohol oxidase) that allows the degradation of some recalcitrant substances like aromatic compounds.^44^

The evolution of the recorded temperatures in the nonbioaugmented pile and the bioaugmented pile is illustrated in Figure 3. In the first 5 days, the temperature ranged between 55 and 62 °C, reaching thermophilic temperature conditions. The maximum temperature, i.e., 63 °C, was observed after 8 days, decreasing to temperatures around 45 °C at day 30 in both piles. After the removal of the semipermeable cover, irrigation, turning, and rebioaugmentation on day 30, the temperature in both piles increased again, reaching the maximum temperature at 33 days with 73 °C (Figure 3). This significant increase of temperature after semipermeable cover removal could be attributed to the environmental conditions and a lack of water in the pile. That may compromise the degradation of organic matter. After irrigation, a reactivation of microbial activity was performed, producing an increase in the temperature. From day 49, the temperature remained close to 45 °C up to day 85. The average ambient temperature recorded at the Guadix meteorological station (Granada) showed maximums of 32 °C and minimums of 16 °C during the operation time. Microbial analysis confirmed that *Salmonella* spp. was not detected at the end of the composting process in either of the two compost piles. However, there was detected a low concentration of *E. coli*, which probably comes from an external pollutant source since was an isolated case detected considering the piles was performed under real conditions and exposed to environmental and natural events. As mentioned above, maintaining the temperature in a thermophilic range was enough to allow the removal of pathogens in the final compost.^32^

**Figure 3:**
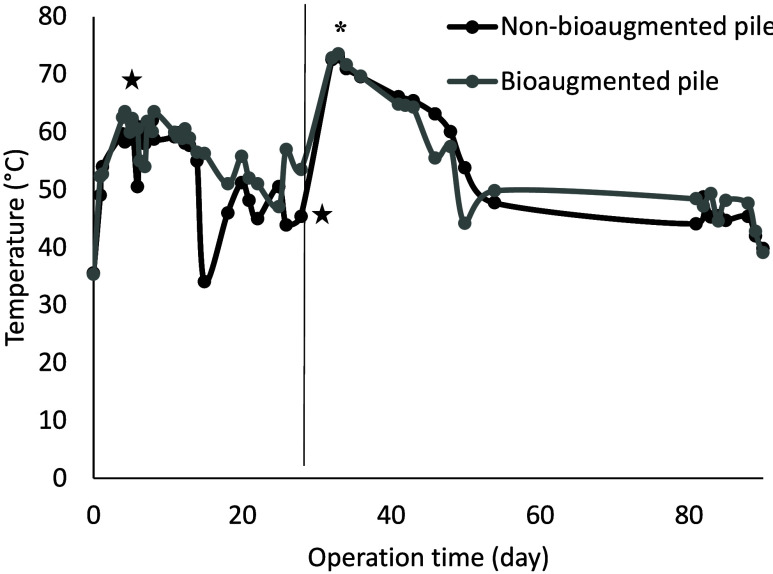
Temperature evolution during the 90 days of composting process in the nonbioaugmented pile and the bioaugmented pile. (★) Bioaugmentation with *P. eryngii*, (--) turning and irrigation, (*) maximum temperature reached, and (−) removal of semipermeable cover.

The physicochemical characterization throughout the composting process is shown in Table 6. It can be observed that the pH remained almost constant in both composting piles, with values around pH 7. Regarding the conductivity of both piles, a slight decrease from 1800 to 1600 μS/cm was observed. As in stage I, both pH and conductivity values remained within the described optimal ranges for composting.^33^ The humidity remained constant around 40% during the first 30 days in which composting was carried out under a semipermeable cover. However, the humidity was reduced to 10% on day 90 in both piles. The TOM content in dry weight showed a decrease from 83.5 to 81.6% in the nonbioaugmented pile, while no variation was observed in the bioaugmented pile (Table 3). Previous studies have reported TOM losses ranging from 15 to 60% under different composting conditions of olive mill solid waste.^28,45^ However, similar minimal TOM losses have been observed in previous research on the composting of sewage sludge.^46^

**Table 6:** Physicochemical Properties during Stage II of the Composting Process with Their Standard Deviation (±)^a^

nonbioaugmented pile	*t*_initial_	15 day	30 day	45 day	90 day
pH	6.9 ± 0.0	6.7 ± 0.1	6.7 ± 0.2	7.4 ± 0.1	6.9 ± 0.1
EC (dS/m)	1.8 ± 0.3	1.7 ± 0.1	1.6 ± 0.0	1.4 ± 0.1	1.6 ± 0.1
humidity (% w/w)	47.6 ± 0.2	40.6 ± 1.3	34.7 ± 2.1	17.8 ± 1.1	10.4 ± 0.3
TS (% w/w)	52.3 ± 0.6	59.7 ± 1.5	65.7 ± 2.1	82.3 ± 1.5	89.7 ± 0.6
MS (% w/w)	8.7 ± 0.6	8.8 ± 0.7	9.9 ± 0.4	17.7 ± 2.2	16.5 ± 0.8
VS (% w/w)	43.3 ± 0.6	50.7 ± 0.6	55.3 ± 1.5	63.0 ± 3.5	73.0 ± 1.0
TOM dry weight (% w/w)	83.5 ± 1.1	85.2 ± 1.0	84.8 ± 0.5	79.3 ± 1.0	81.6 ± 1.0
C (% w/w)	25.3 ± 0.4	29.4 ± 0.6	32.2 ± 1.0	39.2 ± 2.6	42.4 ± 0.6
N (% w/w)	0.9 ± 0.1	1.2 ± 0.0	1.3 ± 0.1	1.8 ± 0.0	2.1 ± 0.1
C/N	27.3 ± 2.2	25.0 ± 0.7	24.4 ± 1.8	21.3 ± 1.6	20.7 ± 1.4
*E. coli* (log CFU/g)					3.7
*Salmonella* spp. (log CFU/g)					n.d
bioaugmented pile	*t*_initial_	15 day	30 day	45 day	90 day
pH	6.9 ± 0.0	6.8 ± 0.1	6.7 ± 0.2	7.3 ± 0.1	6.7 ± 0.0
EC (dS/m)	1.8 ± 0.3	1.7 ± 0.0	1.6 ± 0.0	1.3 ± 0.1	1.6 ± 0.0
humidity (% w/w)	47.6 ± 0.2	36.2 ± 0.5	32.5 ± 6.7	23.3 ± 1.4	10.1 ± 0.1
TS (% w/w)	52.3 ± 0.6	63.7 ± 0.6	67.3 ± 6.7	76.7 ± 1.2	90.0 ± 0.0
MS (% w/w)	8.7 ± 0.6	9.7 ± 0.3	10.1 ± 1.2	13.5 ± 1.7	15.2 ± 1.2
VS (% w/w)	43.3 ± 0.6	54.0 ± 1.0	57.3 ± 5.8	63.3 ± 0.6	75.0 ± 1.0
TOM dry weight (% w/w)	83.5 ± 1.1	84.6 ± 0.4	85.0 ± 1.2	82.4 ± 1.9	83.1 ± 1.2
C (% w/w)	25.3 ± 0.4	31.4 ± 0.3	33.3 ± 3.4	36.6 ± 0.3	43.3 ± 0.6
N (% w/w)	0.9 ± 0.1	1.3 ± 0.0	1.4 ± 01	1.8 ± 0.0	2.0 ± 0.0
C/N	27.3 ± 2.2	23.3 ± 0.4	23.9 ± 0.8	20.3 ± 0.4	22.1 ± 0.2
*E. coli* (log CFU/g)					4.1
*Salmonella* spp. (log CFU/g)					n.d

a n.d. Not detected, conductivity (EC), total solids (TS%), mineral solids (MS%), volatile solids (VS%), total organic matter of dry sample (TOM%), total organic carbon (C%), total nitrogen (N%).

The results obtained for the evolution of the percentages of carbon and nitrogen throughout the process are shown in Table 6. The values in both piles followed a similar trend, with a gradual increase in nitrogen, from 0.9 to 2.1% and 2.0% for the nonbioaugmented pile and bioaugmented pile, respectively. Also, concerning the carbon content, similar upward trends were shown for both piles, rising from 25 to 42–43%. The increase in the percentage of both parameters was closely related to the decrease in humidity content after 45 days. The C/N ratio decreased over time, reaching values of 20 in the nonbioaugmented pile and 22 in the bioaugmented pile.

It is noteworthy that, as described for stage I, calcium was the predominant macronutrient (Figure S2), reaching values close to 4.5% in the nonbioaugmented pile and 3.8% in the bioaugmented pile. However, its proportion was lower than in stage I due to the lower calcium content in poultry manure, which was 9.9 ± 1.1% (w/w) in poultry from stage I compared to 2.1 ± 0.1% (w/w) in stage II. Potassium showed percentages around 2% at both piles. Phosphorus, magnesium, and sodium at the end of the composting process had percentages below 1% in both piles. Finally, the NPK ratio was 2.1/0.3/1.7 for nonbioaugmented pile and 2.0/0.2/1.7 for bioaugmented pile.

The measured concentrations of different heavy metals are listed in Table 7. In the initial mixture (day 0), a high concentration of Zn and Cu is observed, as described in stage I, probably derived from olive leaf pruning. It should be noted that at the end of the composting process, after 90 days, Zn was reduced by 37% in the nonbioaugmented pile and by 57% in the bioaugmented pile. At the end of the composting process, the total heavy metal concentration decreased by 31% in the nonbioaugmented pile and 50% in the bioaugmented pile. This decrease in the heavy metal concentration of the final compost was expected due to the mobilization of some metals due to their interaction with some organic compounds generated during the composting process, such as the humic acids.^34^

**Table 7:** Characterization of Heavy Metals Expressed in mg/kg of the Nonbioaugmented Pile and the Bioaugmented Pile in the Initial and Final Mixture of the Composting Process with its Standard Deviation (±)^a^

	nonbioaugmented pile		bioaugmented pile	
	initial	final	initial	final
Hg (mg/kg)	<0.1	<0.1	<0.1	<0.1
Pb (mg/kg)	2.4 ± 0.6	1.7 ± 0.1	2.4 ± 0.6	1.5 ± 0.4
Ni (mg/kg)	7.6 ± 1.1	4.8 ± 0.4	7.6 ± 1.1	3.7 ± 0.3
Cd (mg/kg)	<0.5	<0.5	<0.5	<0.5
Cr (mg/kg)	12.0 ± 2.6	5.8 ± 1.4	12.0 ± 2.6	6.0 ± 2.2
Zn (mg/kg)	154 ± 4	97 ± 5	154 ± 4	66 ± 14
Cu (mg/kg)	48 ± 40	44 ± 5	48 ± 40	35 ± 6
Cr (VI) (mg/kg)	n.d	n.d	n.d	n.d

a n.d: Non detected.

Figure 4 shows a clear decrease in total phenols, with a reduction of 56% being observed and values close to 0.2% of total phenol content being reached after 90 days of the process. These final values were higher than that described at the end of the poultry-based pile in stage I. However, it is important to note that the initial concentration of total phenols content in stage II was 30% higher than that in stage I, with values of 6.0 ± 0.1 g gallic acid eq/kg of sample and 4.2 ± 0.3 g gallic acid eq/kg of sample, respectively. The higher initial total phenol content would explain the lower removal efficiency observed at this stage II with respect to the stage I, i.e., 56 and 75%, respectively. These results suggested that, although phenols can be used by the microorganisms for anabolic or catabolic purposes,^47^ their concentration in the substrates should be carefully monitored, since their antimicrobial potential would hinder the microbial activity and affect both organic matter conversion and process kinetics.^48^ The phytotoxicity index expressed as a percentage of germination of cress seeds from the compost obtained in each compost pile is shown in Figure 4. Growth of less than 20% compared to the control was observed until day 60 and is related to a high total phenols content, greater than 3 g of gallic acid eq/kg of sample. These findings indicate that the degradation of phenolic compounds, rather than the composting duration, is one relevant factor in obtaining nonphytotoxic compost. In stage II, the temperature suffered an increase after semipermeable cover removal. It could indicate that the biodegradable matter was not totally degraded at that time As well, the pile maintained high mesophilic temperature (about 45 °C) until the end of the process, probably indicating a partial organic matter under a degradation process. This matter may include some phytotoxic compounds that may contribute to the high phytotoxicity reached at 45 days compared to that of stage I.

**Figure 4:**
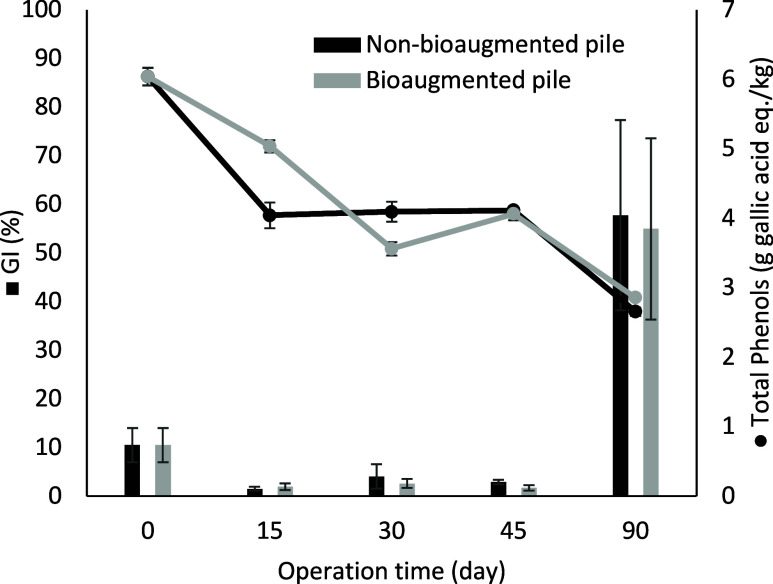
Germination index (GI%) (represented by bars) and total phenol content (depicted by points) for the nonbioaugmented pile and the bioaugmented pile. Error bars represent the standard deviation of triplicate measurements.

The demonstration-scale composting of 2P-OMSW with manure and olive leaves, using forced aeration and a semipermeable cover, proved to be an effective technology, significantly reducing the composting time and improving efficiency. Manure remains the preferred amendment, with no important differences observed between poultry and cow manures in the composting performance. It means that the application of manure source can be adapted according to the available resources of the facilities, making this practice more accessible for its implementation in olive oil factories. Bioaugmentation with *P. eryngii* showed a reduction in heavy metals (Zn and Cu) and a slight decrease in phytotoxicity, suggesting a potential benefit for compost quality regarding downstream application and environmental safety. Despite these slight changes, bioaugmentation did not provide clear advantages, likely due to native microorganisms that specialized during the composting process and were capable of degrading recalcitrant compounds.

## Supplementary Material



## Notes

Figure S1. Evolution of macronutrients expressed in % w/w in the poultry-based pile (a) and cow-based pile (b). Error bars indicate the standard deviation of the triplicate performed. Figure S2. Evolution of macronutrients expressed in % w/w in the non-bioaugmented pile (a) and bioaugmented pile (b). Error bars indicate the standard deviation of the triplicate performed. (PDF)
