# Supplementary material for: Assessment of Different Strategies for Composting of the Two-Phase Olive Mill Solid Waste: A Demonstrative Scale
Source: ACS Agric Sci Technol. 2025 Aug 11;5(9):1869–78. doi: 10.1021/acsagscitech.5c00286 (PMC12442762; doi:10.1021/acsagscitech.5c00286)
Supplement: Supplementary file 1 [file as5c00286_si_001.pdf]

Assessment of Different Strategies for Composting of the Two-phase Olive Mill Solid Waste: A Demonstrative Scale

Sara Velilla-Delgado[†,††,‡], Juan Cubero-Cardoso[†,††,‡], Antonio Serrano[†,††], Elisabet Aranda[†,††], Concepción Calvo[†,††], Tatiana Robledo-Mahón[†,††,*]

[†] Environmental Microbiology Group, Institute of Water Research, University of Granada, Espacio V Centenario, Avenida Madrid 11, 18012 Granada, Spain.

[††] Department of Microbiology, Pharmacy Faculty, University of Granada, Campus Universitario Cartuja s/n, 18011 Granada, Spain.

[‡] These authors contributed equally

[*]Corresponding author: Tatiana Robledo-Mahón (trobledo@ugr.es)

Suplementary Material

List of items

Figure S1. Evolution of macronutrients expressed in % w/w in the poultry-based pile (a) and cow-based pile (b). Error bars indicate the standard deviation of the triplicate performed.

Figure S2. Evolution of macronutrients expressed in % w/w in the non-bioaugmented pile (a) and bioaugmented pile (b). Error bars indicate the standard deviation of the triplicate performed.

(a)

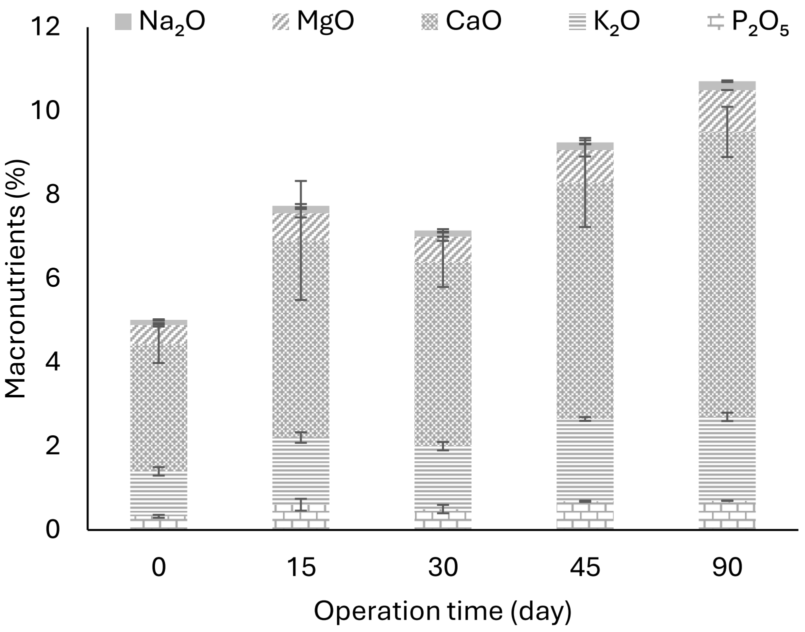

(b)

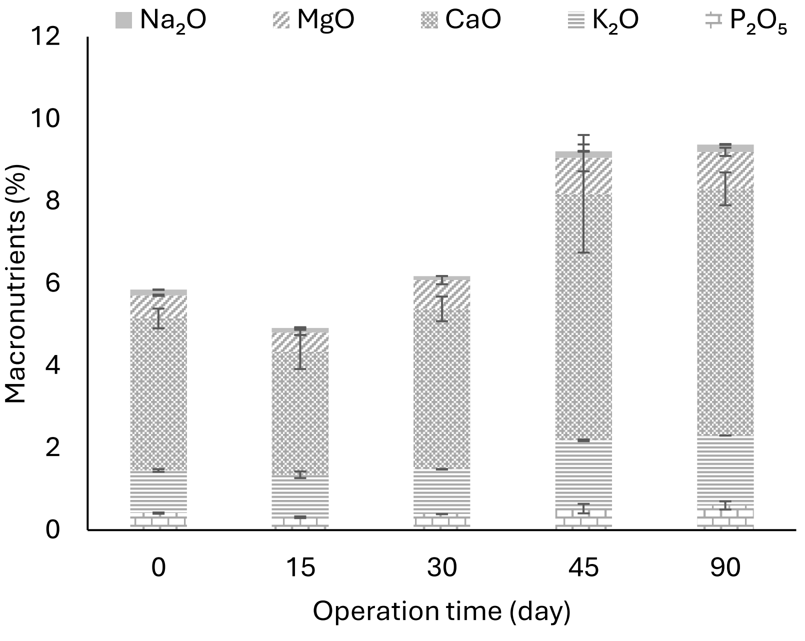

Figure S1. Evolution of macronutrients expressed in % w/w in the poultry-based pile (a) and cow-based pile (b). Error bars indicate the standard deviation of the triplicate performed.

(a)

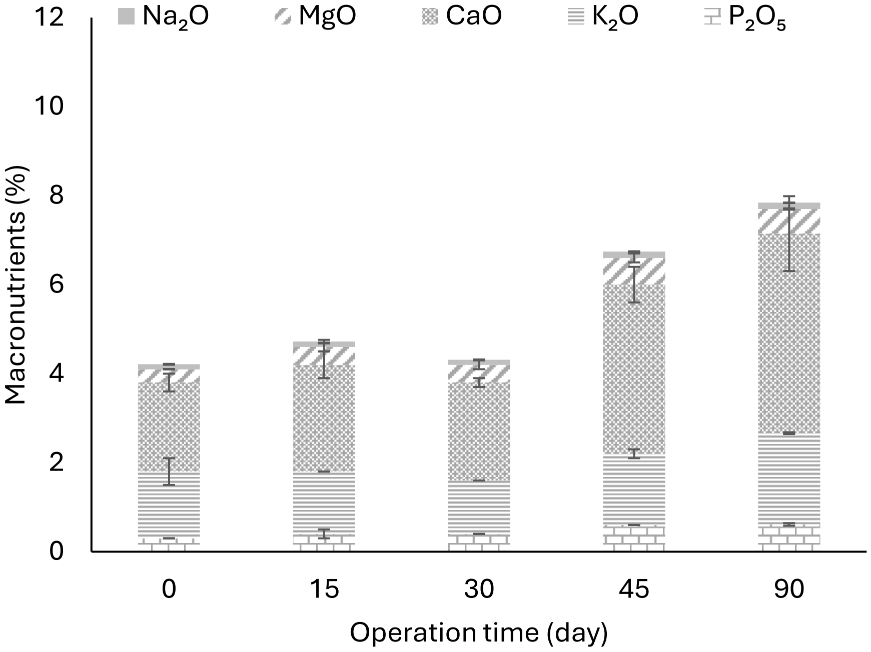

Na2O
MgO
CaO
K2O
P2O5
12
10
8
6
4
2
0
Macronutrients (%)
0
15
30
45
90
Operation time (day)

(b)

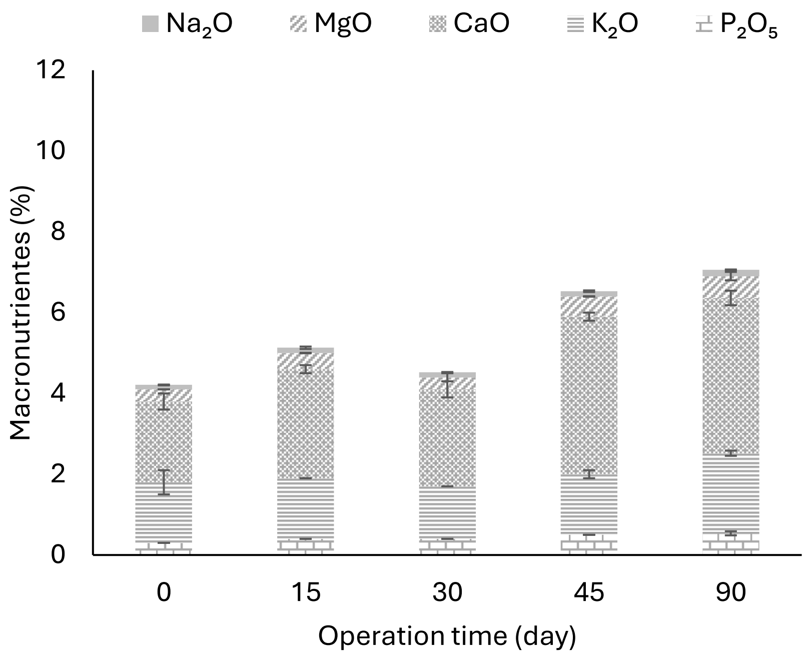

Figure S2. Evolution of macronutrients expressed in % w/w in the non-bioaugmented pile (a) and bioaugmented pile (b). Error bars indicate the standard deviation of the triplicate performed.
